# The Perceptions of Domestic Violence by a Family Member Who Uses Crack or Cocaine: A Secondary Retrospective Cross-Sectional Study

**DOI:** 10.3390/ijerph19106325

**Published:** 2022-05-23

**Authors:** Gilmar Manoel de Barros, Alessandra Diehl, Adaene Alves Machado de Moura, Adriana Inocenti Miasso, Ronaldo Laranjeira, Cláudio Jerônimo da Silva, Sandra Cristina Pillon, Christopher Wagstaff, Ana Lucia de Moraes Horta

**Affiliations:** 1 Programa Recomeço, School Paulista of Nursing, Federal University of São Paulo, Sao Paulo 04024-002, Brazil; gilmar.barros.psico@gmail.com (G.M.d.B.); analuciahorta18@gmail.com (A.L.d.M.H.); 2 Psychiatric Nursing and Human Science Department, Faculty of Nursing at Ribeirão Preto, University of São Paulo, Sao Paulo 14040-902, Brazil; alediehl@terra.com.br (A.D.); adaene_moura@usp.br (A.A.M.d.M.); amiasso@eerp.usp.br (A.I.M.); 3 Department of Psychiatry, Federal University of Sao Palo (UNIFESP), Sao Paulo 04038-020, Brazil; laranjeira@uniad.org.br (R.L.); claudio.jeronimo@uol.com.br (C.J.d.S.); 4 School of Nursing, Institute of Clinical Sciences, College of Medical and Dental Sciences, University of Birmingham, Birmingham B15 2TT, UK; c.wagstaff@bham.ac.uk

**Keywords:** domestic violence, cocaine use, family, substance-related disorders

## Abstract

Objective: To evaluate the relationship between crack/cocaine use and domestic violence perpetration from the perspective of substance users’ families. A secondary retrospective cross-sectional study, with 3162 family members of crack/cocaine users seeking treatment in the Recomeço Família Programme in São Paulo/Brazil was undertaken. Family members of crack/cocaine users reported that their relatives were more involved in domestic violence such as stealing (money and objects) at home [Odds Ratio Adjusted ORA = 2.17 (CI 95% 1.87; 2.53)], the family gave money to the user to buy drugs [ORA = 1.27 (1.08; 1.48)], and having problems with the judiciary [ORA = 1.48 (CI 95% 1.28; 1.71)]. Relatives of snorted cocaine users reported that there was physical and interpersonal violence, such as fathers being assaulted [ORA = 2.50 (CI 95% 1.08; 5.82)], assaulted someone else [ORA = 1.86 (CI 95% 1.32; 2.60)], threats of violence fights, arguments when the family talk about problematic drug use [ORA = 1.50 (CI 95% 1.13; 1.96)] and threatened some family members [ORA = 1.52 (CI 95% 1.14; 2.04)]. In this sample, there was a connection between crack/cocaine use and the perpetuation of domestic violence, corroborating with important implications for public policies, substance use treatment and prevention of domestic violence interventions.

## 1. Introduction

There is a strong association between substance abuse and domestic violence [1,2], with an impact on the social, economic, physical, psychological and health of victims [3]. In Brazil, every two minutes, a woman is a victim of domestic violence [4]. While in the United States, domestic violence affects around 10 million people every year; as many as 1 in 4 women and 1 in 9 men; 92% of men who assaulted their female partner had used substances on the day of the assault; 67% had used both cocaine and alcohol [5]. However, there is still a paucity of empirical research on the role of other substances in domestic violence, especially between the two main *routes* of cocaine *used* (smoked and snorted) [6], particularly, within Latin American countries, such as Brazil, which is the largest market for cocaine use in South America with approximately 1.5 million users [7,8]. Some Brazilian studies that examined crack/cocaine users have shown that regarding social and legal problems, about 97% of crack/cocaine users reported some involvement with violence. Eighty four percent (84%) reported some kind of drug-related violence. In the majority of these cases, the violent act was initiated by the user [9]. Crack users present a significantly higher rate of occupational, family, and legal problems and reported more illegal and violent activities such as burglary and theft (23%) and threatening or assaulting (32%) than non-cocaine users [10]. Among crack users in the downtown area of São Paulo popularly known as “cracolândia”, around 87% do not have any paid activity, of these, almost 80% have lived this way for at least one year and 56.8% reported not having any source of income, whether from work or income transfer programs. There is a difficulty in estimating the value of the drug by users, however, a national study states that people who circulate in the region spend, on average, R$ 192.59 (1 real = 4.62 dollars) per day with crack [11].

It is important to note that domestic violence is a term that refers not only to the act of physical abuse but also to, “any incident or pattern of incidents of controlling, coercive, threatening, violent or abusive behavior”, including but not limited to, “psychological, physical, sexual, financial and emotional abuse”. Thus, domestic violence is not referred to exclusively as intimate partner violence (IPV) as commonly found in the research, since domestic violence can also be directed at children, parents, or any other relative, regardless of age, gender, sexual orientation, or ethnic origin [12]. In many instances, family members find themselves victims of theft, as substance users resort to selling their property in exchange for the desired substance [13,14,15]. Family members may also be forced to compensate financially for debts collected because of substance use, or due to an inability of the substance user to go to work [10]. Those living with a substance user may be compelled to also take time away from work to cope with stress or stress-related illness. Additionally, family members may find themselves victims of threats due to debt acquired by substance users [14].

Hence the importance of obtaining data from the perspective of the families of substance users, who often experience or witness domestic violence and to the social, psychological, and financial pressures on families [16,17]. In addition, family data can help predict the development of greater health disparities in the country. Additionally, such research can help to maximize the effects of treatment and rehabilitation processes on the substance users’ families to increase and improve services aimed at supporting the health and well-being of the population that lives with substance users and suffers the domestic violence caused by them [18,19].

In Brazil, systematic information on the profile of domestic violence in this population is scarce. Nevertheless, one of the few studies that investigated family members of substance users (the most commonly used drugs were cannabis, cocaine and crack-cocaine) had a family member from 3153 families with a substance user from the five geographic regions in Brazil; revealing that 35.9% of family members have been threatened by the substance using relative and 36.4% had been physically assaulted by the substance using relative [18]. Mothers or wives/girlfriends of substance users reported a greater impact on the family, showing both psychological and physical symptoms and using avoidance as a coping mechanism [19]. Consequently, the association between crack/cocaine use and domestic violence has important implications for governmental policy, substance use treatment and domestic violence interventions [20].

This study aimed to evaluate the relationship between crack/cocaine use and domestic violence perpetration from the perspective of substance users’ families. 

## 2. Material and Method

### 2.1. Study Design

This is a cross-sectional study conducted based on secondary data from family members who sought outpatient treatment for substance dependence in all 14 units of the Recomeço Família Program, located in the municipalities of São Paulo, Campinas, Guarulhos, Francisco Morato, Jundiaí and Ferraz de Vasconcelos in the State of São Paulo, Brazil. It is a representative sample of families that usually seek support at this program obtained from a total of 5201 (100%) records of family members, who had sought treatment at the respective services.

The Recomeço Família Program is part of the Recomeço Program, which aims to provide psychological support and guidance to family members based on the “codependency approach”, similar to the 12-step models for families attending mutual support groups, such as Al-anon and Nar-anon, Al-teen. It is a multi-secretariat program by the Government of the State of São Paulo, implemented in partnership with the State Secretariats of Health, Justice and Citizenship with 11 Citizenship Integration Centers, located in the city of São Paulo, Brazil, and five surrounding cities.

The eligibility requirements for the study were the medical records and engaging with the Recomeço Família Program for the first time. The data was collected over one month and involved examination of medical records from 2014–2018 by health care professionals, who had been trained in the data collection process. As this study was just interested in cocaine and crack use only 3162 (68.8%) families were included to create the sample for the study.

### 2.2. Instruments

#### 2.2.1. Socio-Demographics Information

Gender, marital status (single, union stable, divorced/separated, widowed), living with partner, homeless, occupational status (employed, unemployed or retired), literate (those who know how to read and write, had the necessary knowledge of reading and/or calculations to have a socially functional life were considered literate) and living with the substance user The nature of the kinship of the person who sought treatment: Father/mother or guardian, spouse, or another family member (brothers, uncles, and grandparents) [15].

#### 2.2.2. Substance Use

Cocaine use (snorted or smoked) was individually evaluated in the preceding three months, with dichotomous responses for each variable (Yes/No) [16,21].

#### 2.2.3. Information about Domestic Violence Perpetrated by Substance User 

These variables were evaluated based on whether violence was physical/interpersonal or non-physical, whether the family sustained the substance use with money to buy drugs in the last 12 months, and whether the substance user had assaulted someone who was not a family member. Participants were also asked which family member had been victimised by the substance user and whether conversations relating to substance use subsequently resulted in threats or violence after a talk regarding the problems related to drug use. For each question, dichotomous responses were given (Yes/No) [15,21].

### 2.3. Ethical Issues

The study was approved by the Research Ethics Committee of the Federal University of São Paulo (UNIFESP) (Process No. 90411318.2.0000.5505).

### 2.4. Statistical Analysis

The data was first organised into a spreadsheet using MS-Excel^®^, and double typing was performed as a way of checking for accuracy and consistency. The data was then evaluated using Statistical Package for Social Sciences (SPSS) V. 26.0 for Windows, Chicago, IL, USA. Exploratory analysis of the data was conducted through frequencies (*n*) and percentages (%). The Chi-squared test was carried out to test the significance of the relationship between the qualitative variables, as well as to compare possible differences between observed and expected frequencies for each event. Thus, variables that did not reach levels of significance were not included in the later stage, to include a greater number of variables. Exponentiated coefficients were interpreted as odds ratios adjusted (ORA) in the multivariate analysis. Snorted and smoked cocaine use (independent variable) and covariates (sociodemographic (age of group; marital status; literate; occupational status, homeless and living with the substance user) and acts of violence committed by the substance using family member (physically assaulted someone in the family; threats a family member; assaulted someone else; threats or violence after a talk about the problems related to drug use; the family gave money for the user to buy drugs; stole objects or money from home or problems with the judiciary, and father or mother assaulted by drugs user) were used. For all tests, a significance level of *p*-value < 0.05 with a 95% confidence interval (CI) was considered.

## 3. Results

### 3.1. Sample Characteristics

Table 1 shows the sociodemographic characteristics of the family members (*n* = 3162) with a substance user, divided into two groups of users, with cocaine being the main drug used, by the following routes [smoked 1378 (43.6%) and snorted 2633 (83.3%)].

Regarding the characteristics of family members who sought assistance in these services, there is a predominance of a group of women (81.4%), adults aged (50–59 years) (23.4%), married (46%), illiterate (65.3%), employed (60.2%) and who lived with a drug user (71.9%).

In the total sample men were predominant (79.5%), young people (18–20 years) (37.8%) and adults (30–39 years) (31.0%), single (33.3%), although 46% of the records did not contain this information, slightly more than half were literate (58.7%), employed (53.4%) and only 7.4% was homeless (data not available in table).

The results show the differences between sociodemographic characteristics according to the route of cocaine use (smoking or snorted), which shows two heterogeneous groups. Amongst crack users half are women, adults (age groups 30 to 59 years), mostly widowers, illiterate and who were homeless. While cocaine users were mostly men, young (<29 years), married and not homeless.

### 3.2. Substance Use and Sociodemographic Characteristics

In multivariate analyzes, the highest odds ratios for crack use were among women [OR Adjusted (ORA) = 1.52 (Confidence interval (CI) 95% 1.15; 2.00)], adults [30–39 years old (ORA = 3.94 (CI 95% 2.48; 6.26))], potentially among users with 40–49 years old [ORA = 5.17 (CI 95% 3.09; 8.64)] and >50 years old [ORA = 3.86 (CI 95% 2.08; 7.16)]. Risks almost doubled among the unmarried [ORA = 1.78 (CI 95% 1.36; 2.32)] and unemployed [ORA = 1.57 (CI 95% 1.20; 2.06)], illiterate [ORA = 2.47 (CI 95% 1.50; 4.08)] and homeless [ORA = 7.38 (CI 95% 4.07; 13.37)]. Data available in the Table 2.

This group differs from cocaine users, 84% male, younger (<17 to 29 years), married, literate, and worked but there was also a considerable group that was retired and 65% homeless. In the multivariate analyzes, the highest odds ratios for the snorted cocaine use were three times or greater among younger individuals [age group <18 (ORA = 3.90 (CI 95% 1.76; 8.64) and 18–29 (ORA = 3.55 (CI 95% 2.01; 6.26)]. Beside not been homeless [ORA = 3.48 (CI 95% 1.92; 6.29)] as well as risks doubled among married people [ORA = 2.12 (CI 95% 1.43; 3.15)] and literate [ORA = 2.27 (95% CI 1.34; 3.87)] (Table 2).

### 3.3. Domestic Violence and Cocaine Use

Table 3 shows that more than half of the relatives of cocaine users (smoked) experienced domestic violence, 49.6% gave money to their loved one for drug use, and 52.7% had been stolen from by the substance user (objects/money) at home and 51.1% of the drug users have had problems with the justice system. In the multivariate analysis, families with cocaine users (smoked) were more likely to have given money for drug use [ORA = 1.27 (CI 95% 1.08; 1.48)], to have been stolen (money/objects) at home [ORA = 2.17 (CI 95% 1.87; 2.53)] and having a member with problems in court [ORA = 1.48 (CI 95% 1.28; 1.71).

Unlike the group of family members with a member who smoked cocaine, who differed considerably as a group, the families who had a member who snorted cocaine were more like to experience domestic violence. However, the families with a member who snorted cocaine had a 44.0% less chance of having objects/money stolen, and the family member was 28.0% less likely to have legal problems.

The fathers were the main victims of aggression [yes 83 (93.3%) versus no 6 (6.7%), *p* = 0.032] and double odds ratios [ORA = 2.50 (CI 95% 1.08; 5.82)] of being victims of aggression by cocaine (snorted) users. Among fathers, the odds ratios were low (47.0%) of being victims of aggression by smoked cocaine users (Table 3).

### 3.4. Physical and Interpersonal Violence and Cocaine Use

Table 4 shows an inversion in the types of violence (physical and interpersonal) with higher percentages and odds among family members of snorted cocaine users, with threats to someone in the family [ORA = 1.52 (CI 95% 1.14; 2.04)], threats to other people [ORA = 1.86 (CI 95% 1.32; 2.60). When the family members had tried ry to talk about the problem, 90.0% of the users react with threats or violence [ORA = 1.50. (CI 95% 1.13; 1.96)]. In the group of family members of crack users, the odds ratios were ORA 1.25 (CI 95% 1.07; 1.45) physically assaulted someone in the family. The odds ratios were lower (19%) among family members of smoked cocaine users; the arguments, threats and fights were lower among smoked cocaine users after their families tried to talk to them about the problems associated with their drug use (Table 4).

## 4. Discussion

The main finding of this study corroborates the links between crack-cocaine use and the perpetration of both physical and non-physical domestic violence. More than 50% of participants reported that their family members had committed thefts at home, associated with both forms of cocaine. Physical assaults on relatives of crack using family members were reported by more than a third of the participants. More than a quarter of the participants reported that users had threatened a family member, which was linked to the use of cocaine (snorted).

### 4.1. Sociodemographic Characteristics

When examining the sociodemographic characteristics of the family members and those reporting to have experienced domestic violence, it is noted that the family caregivers were female, adults, with a low level of education and the substance users, who frequently lived with their parents. It was been noted that crack users were younger and had lower levels of education; the presumption is that crack use impacts the central nervous system, resulting from crack use starting at an earlier age, being linked to social circles, the unravelling of family bonds, involvement with crime and violence, poor school performance and eventually dropping out of school [13,15,19].

Our sample corroborates the statistics of domestic violence within Brazilian society. Additionally, the literature surrounding substance use treatment and therapy, shows the family play a role in aiding the treatment process [22]. Working with family members of substance users as a treatment intervention, as happens in the Programa Família Recomeço, is a useful component in helping the users maintain the motivation to receive treatment. This study corroborates with the literature showing the family as a source of care and support, allowing users to progress towards recovery [23].

### 4.2. Domestic Violence and Cocaine Use

#### Non-Physical Domestic Violence and Problems with the Judiciary

Results indicated that theft of money or objects from the home was the form of non-physical domestic violence most frequently reported by participants. These findings suggest that crack cocaine users typically steal from family members as a way of sustaining their drug use, a characteristic of financial domestic abuse. Globally, substance use has been mentioned in almost all reports to police about domestic violence. Acquisition crimes such as stealing have been evidenced to be associated with crack cocaine use more so than the use of any other substance [24]. In a qualitative study, crack cocaine users from São Paulo reported repeatedly stealing from their families to fund their substance use [25]. Further literary evidence substantiating our findings derives from the research of Chaves et al. [26] (2011). Here, we present compelling evidence to suggest that crack cocaine users habitually steal, from their homes and their family members, for money to purchase crack cocaine.

High-frequency use also substantiates the association between snorted cocaine use and theft from the home. Users of snorted cocaine characteristically display compulsive patterns of use [27] and drug-seeking behaviours [28], in keeping with Goldstein’s economic-compulsive model [29]. This model suggests that individuals who frequently engage in the use of substances perpetrate profit-orientated crime to finance this substance use disorder. Additionally, heavy snorted cocaine users are 3.5 times more likely to perpetrate acquisition crimes to obtain cocaine than infrequent and non-cocaine users [30], affirming a link between compulsive snorted cocaine use and theft.

Results of this study confirm that both routes of crack cocaine use are associated with involvement with the law, suggesting that cocaine users often commit crimes, and thus encounter legal trouble. A compelling relationship between substance use, and crime perpetration has been identified throughout research [31,32], however the nature of this relationship remains unclear. Primarily, studies have evidenced substance use to cause crime perpetration. Cocaine use specifically has been shown to lead to sex-related offences, violent crimes, involvement in drug crimes, and income-generating offences, as previously mentioned [9,33,34].

### 4.3. Physical and Interpersonal Domestic Violence

There are important points to take into consideration when discussing the violence within the family in this study. Firstly, a considerable percentage of the women used both forms of cocaine, and physical domestic violence was twice as likely amongst female crack users. Additionally, mothers had a higher odds ratio of being assaulted by their drug-using children. Conversely, 81.4% of the people who had sought help through the Programa Recomeço Familia were women. Similarly, a South African study demonstrated that substance use was significantly associated with female perpetration of IPV [35]. A systematic review [36] investigated the link between cocaine use and the perpetration of IPV in patients undergoing substance abuse treatment. In some cases, these samples included patients who were receiving alcohol abuse treatment, suggesting that polysubstance use is an indicating factor in IPV. The association between cocaine use and IPV was also found in some community studies [36]. Substance use has also been evidenced to be a consequence of domestic violence. Victims of physical domestic violence frequently engage in substance use to allow them to cope with the abuse they are experiencing [14,37]. Child abuse victims also commonly report substance use in later life [38]. Although these studies are not specific to cocaine use, they demonstrate that substance use not only leads to domestic violence but simultaneously being a victim of domestic violence can also lead to substance use.

The most frequent form of physical and interpersonal domestic violence reported in this research was a physical assault of a family member; indicating that physical aggression and hostility towards family members are behaviours consistent among cocaine users. A systematic review and meta-analysis [39] on diverse health outcomes in cocaine and crack users, found moderate evidence for an association between cocaine use and violence. A previous study [40] has shown that Brazilian crack and snorted cocaine users had more family problems as compared with users of other substances, and cocaine use was associated with more severe violence [41]. The current study also investigated assaults on people outside of the family, to further establish the links between cocaine use and physical violence. Our results suggest that cocaine users were more likely to assault a member of their family than they were someone outside of the family. Goldstein’s [42] (1991) study into the frequency of cocaine use and violence partially vindicates these findings, showing a higher frequency of violence perpetration by female cocaine users towards a spouse or partner than a stranger or acquaintance.

Our results indicated that crack cocaine use was associated with physical assaults on a family member, whereas snorted cocaine use was associated with threatening a family member. These findings are corroborated by Brazilian literature showing that crack cocaine users exhibit more problematic and violent family relationships than other substance users [43,44]. More severe violence among crack users is reflective of the increased severity and intensity of psychoactive effects contributing to heightened impulsivity and risk taking [45]. As a result, behaviours that users may not necessarily exhibit whilst sober, such as perpetration of physical violence towards a family member, they now are predisposed to when under the influence of crack cocaine. Vaughn and colleagues [6] demonstrated that American crack cocaine users were more physically violent towards family members than users of snorted cocaine, further validating our findings. However, Vaughan and collaborators highlighted that whilst crack cocaine users were more likely to be violent this was related to sociodemographic characteristics and psychiatric variables as opposed to the nature of the substance [6].

Contrastingly, despite both crack and snorted cocaine users being violent after conversations about the problems related to their use, these prevalence rates were much lower than the prevalence of physical assaults on, or threats towards, family members. Thus, other existing factors must trigger such behaviour, such as the assertion of power and control, communication difficulties, and expression of negative emotions (e.g., anger or stress) [46]. These triggers in combination with a predisposition to aggression elicited by cocaine may account for the physical assault and threatening behaviour reported by participants. As the present study did not fully investigate the causes of domestic violence, conclusions about this cannot be made, however, future studies may wish to investigate this.

### 4.4. Implications for Clinical Practice

Although this study was carried out in community services, training is strongly recommended for all professionals who are frequently in contact with patients who are violent and regular cocaine/crack, and other substances, users. We recommend the use of protocols with screening tools regardless of the setting and complexity of the patient group. For example, Emergency Rooms need a structured response because of the frequency with which they need to deal with people who have conditions that are frequently associated with violence such as narcissistic, antisocial, paranoid and borderline personality disorders, as well as service users with bipolar and schizophrenic conditions.

Whilst this retrospective cross-sectional study highlights that 90% of the people involved in some form of violence were using cocaine, there have not been, to the best of our knowledge any studies which demonstrate how interventions with family communication that can improve this cocaine related violence.

Further, the identification of the links between crack and snorted cocaine use and domestic violence, emphasises the necessity for more domestic violence services and shelters across Brazil, aimed at preventing violence within families of substance users and offering support to families experiencing domestic violence. Additionally, more training and guidance must be given to staff in treatment interventions, such as those at the Recomeço Familia Programme, to identify domestic violence perpetration in initial assessments.

### 4.5. Limitations

We are aware that the question regarding the cocaine user’s involvement with the judiciary is not specific to domestically violent crimes, and therefore we are cautious about interpreting this finding. Crimes or offences perpetrated by cocaine users may not have necessarily been violent or abuse-related, or even substance use-related, and thus may not have any relevance to our study. As a result, we cannot ascertain whether significant associations identified between crack and snorted cocaine use and past involvement with the law are reflective of violent crimes engendered by cocaine use, or due to the unspecific nature of the question. This limitation highlights flaws in the Brazilian National Alcohol and Drugs Survey (BNADS) [8] questionnaire used to interview participants.

Given that this was a cross-sectional study, the research is vulnerable to the methodological limitations of this study design. The prime issue was that the study was restricted in its ability to confirm causality. Thus, any conclusions about the relationship between crack cocaine use and domestic violence are merely associative and remain vulnerable to confounding variables, such as individual traits of personality and comorbidities, that were not investigated. In addition, it is important to point out that, whether experiencing or committing violence, the use of crack-cocaine is not a plausible direct causal agent; rather, crack use likely acts as a facilitator or marker for risky behavior (e.g., shared drug paraphernalia, high-risk sexual activity) or exposure to distinct risk environments (e.g., violent drug markets, sex work) where these consequences commonly occur) [39].

Additionally, as participant responses were collected retrospectively in the original study, our results are susceptible to recall bias. However, the research team mitigated against this by using closed-ended questioning. Further, participants did not answer all questions correctly; therefore, not all findings were accurate and reflective of the cohort. Moreover, this was a retrospective secondary study, had this been a primary or prospective study, missing data could have been accounted for and incorrect data could have been limited.

### 4.6. Future Research

Future studies should investigate crack cocaine use, domestic violence, and the association between the two in multiple outpatient clinics, treatment centres, and hospitals across Brazil and at a community level. Research in other developing nations across South America and other continents, with a high prevalence of cocaine consumption, should also be conducted [6]. Future research also should investigate the prevalence of domestic violence both when under, and not under, the influence of cocaine to determine if there is a causal relationship, and confounding variables. Additionally, further research needs to be done to assess whether the severity of violence is impacted by alcohol consumption in these cases. Such research would provide for greater representativeness and generalisability.

Finally, we recommend that health services invest in shared record systems (primary care services and emergency units), that contain clear descriptions of both public and domestic violence, and the use of psychoactive substances. The use of standardized instruments to assess substance use and violence is of crucial importance, especially in the current post-pandemic era, in which mental health problems and substance use appear to have intensified. All the tools used in this study had been validated for use in Brazilian Portuguese.

Often, the formal preparation of emergency service professionals around these issues is scarce and assistance is directed only to clinical emergencies and the demands of this population cannot be met by emergency services alone. As such, we have a reproduction of a cycle of violence that perpetuates itself, unless care is implemented, particularly in men’s health. A theme that needs to be considered in future studies is the generalised problems caused by both domestic violence and public violence.

## 5. Conclusions

Mental health, violence and substance use remain topics that are socially stigmatized, including amongst health professionals. For both research and clinical practice, these areas are permeated by moral, ethical, and legal issues, frequently leaving this vulnerable population short of more assertive assistance. In this sample, there was an association between cocaine (both crack and snorted cocaine) use and domestic violence, and additionally, threats of violence were more common from snorted cocaine users when families tried to talk about the problems. These findings corroborat with previous research and have important implications for government policies, substance use treatment and domestic violence interventions.

## Figures and Tables

**Table 1:** Sociodemographic characteristics of family members, drug users and the distribution of cocaine (smoked or snorted) in the sample.

		Member [*n* (%)]	Cocaine [*n* (%)]	
		Family (Yes)	Smoked ^ǂ^ (Yes)	Snorted ^ǂ^ (Yes)
Gender	Male	505 (16.0)	1060 (42.1)	2113 (84.0) **
	Female	2574 (81.4)	270 (50.9) *	424 (80.0)
Age of group (years)	<18	83 (2.6)	74 (29.4)	224 (88.9) *
	18–29	413 (13.1)	417 (34.9)	1064 (89.1) *
	30–39	553 (17.5)	524 (53.4) *	757 (77.2)
	40–49	658 (20.8)	221 (52.1) *	340 (80.2)
	50–59	740 (23.4)	71 (53.8) *	99 (75.0)
	>59	556 (17.6)	7 (30.4)	19 (82.6)
	Answer not given	159 (5.0)		
Marital status	Single	776 (24.5)	412 (39.2)	943 (89.6)
	Married	1455 (46.0)	154 (32.0)	433 (90.0) *
	Divorced/separated	408 (12.9)	83 (51.2)	137 (84.6)
	Widowed	261 (8.3)	12 (85.7) *	10 (71.4)
	Answer not given	262 (8.3)	-	-
Literate	Yes	1097 (34.7)	711 (38.3)	1633 (88.0) *
	No	2065 (65.3)	60 (61.2) *	73 (74.5)
	Answer not given	-	-	-
Occupational status	Employed	1904 (60.2)	638 (37.8)	1496 (88.6) *
	Unemployed	536 (17.0)	530 (52.7) *	760 (75.5)
	Retired	340 (10.8)	15 (36.6)	34 (82.9)
	Answer not given	382 (12.1)	-	-
Homeless	Yes	-	194 (82.6) *	154 (65.5) *
	No	-	1062 (39.7)	2275 (85.0)
	Answer not given	-		
Living with the substance user	Yes	2272 (71.9)	-	-
	No	639 (20.2)	-	-
	Answer not given	251 (7.9)	-	-

Notes: *p*-value ≤ 0.001 * and *p*-value < 0.05 ** through ^ǂ^ Chi-square test. Total = Cocaine [smoked (*n* = 1378) and snorted (*n* = 2633)].

**Table 2:** Sociodemographic characteristics of cocaine (smoked and snorted) users reported by family.

		Cocaine Smoked ^¥^		Cocaine Snorted ^¥^	
		OR Crude (CI 95%)	ORA (CI 95%)	OR Crude (CI 95%)	ORA (CI 95%)
Gender	Male	Ref	Ref	1.31 (1.03; 1.66) *	1.20 (0.83; 1.73)
	Female	1.42 (1.18; 1.72) *	1.52 (1.15; 2.00) *	-	-
Age of group (years)	<18	Ref	Ref	2.50 (1.46; 4.30) *	3.90 (1.76; 8.64) *
	18–29	1.30 (0.96; 1.73)	1.50 (0.95; 2.36)	2.56 (1.70; 3.87) *	3.55 (2.01; 6.26) *
	30–39	2.76 (2.04; 3.73) *	3.94 (2.48; 6.26) *	1.06 (0.71; 1.58)	1.20 (0.70; 2.05)
	40–49	2.62 (1.88; 3.65) *	5.17 (3.09; 8.64) *	1.26 (0.81; 1.97)	1.24(0.68; 2.24)
	>50	2.43 (1.60; 3.69) *	3.86 (2.08; 7.16) *	-	-
Marital status ^¥^	Unmarried	1.80 (1.45; 2.20) *	1.78 (1.36; 2.32) *	-	-
	Married	Ref	Ref	1.97 (1.44; 2.70) *	2.12 (1.43; 3.15) *
Literate	Yes	Ref	Ref.	2.52 (1.56; 4.05) *	2.27 (1.34; 3.87) **
	No	2.54 (1.67; 3.85) *	2.47 (1.50; 4.08) *	-	-
Occupational status	Employed	Ref	Ref	1.60 (0.70; 3.66)	-
	Unemployed	1.83 (1.56; 2.14) *	1.57 (1.20; 2.06) *	0.63 (0.27; 1.45)	-
	Retired	0.94 (0.50; 1.80)	0.45 (0.18; 1.10)	-	-
Homeless ^¥^	Yes	7.20 (5.08; 10.16) *	7.38 (4.07; 13.37) *	Ref	Ref
	No	-	-	2.98 (2.23; 3.98) *	3.48 (1.92; 6.29) *
Living with Family	Yes	-	**-**	1.60 (1.28; 1.99) *	0.76 (0.50; 1.15)
	No	2.11 (1.77; 2.53) *	0.98 (0.73; 1.32)	-	-

Notes: *p*-value ≤ 0.001 * and *p*-value < 0.05 ** through ^¥^ Multivariate analysis. ORA = Odds Ratio Adjusted. Statistical analysis controlled by alcohol use, gender and age. Total = Cocaine [smoked (*n* = 1378) and snorted (*n* = 2633)].

**Table 3:** Domestic violence reported by family.

		Cocaine Smoked			Cocaine Snorted		
		Yes ^ǂ^ [*n* (%)]	OR Crude (CI 95%) ^¥^	ORA (CI 95%) ^¥^	Yes ^ǂ^ [*n* (%)]	OR Crude (CI 95%) ^¥^	ORA (CI 95%) ^¥^
The family gave money for the user to buy drugs	Yes	474 (49.6) *	1.42 (1.20; 1.65) *	1.27 (1.08; 1.48) **	778 (81.5)	0.83 (0.68; 1.02)	-
	No	904 (41.0)	-	-	1855 (84.1)	-	-
The drug user stole objects or money from home	Yes	931 (52.7) *	2.36 (2.04; 2.73) *	2.17 (1.87; 2.53) *	1407 (79.6) *	0.54 (0.44; 0.66) *	0.56 (0.46; 0.69) *
	No	447 (32.0)	-	-	1226 (87.9)	-	-
The drug user has had problems with judicary	Yes	653 (51.1) *	1.66 (1.44; 1.92) *	1.48 (1.28; 1.71) *	1021 (79.8)	0.66 (0.55; 0.80) *	0.72 (0.59; 0.87) *
	No	725 (38.5)	-	-	1612(85.6) *	-	-
Father assaulted by drugs user	Yes	25 (28.1) **	0.49 (0.31; 0.79) **	0.53 (0.33; 0.85) **	83 (93.3) **	2.84 (1.23; 6.53) **	2.50 (1.08; 5.82) **
	No	1353 (44.0)	-	-	2550 (83.0)	-	-
Mother assaulted by drug user	Yes	35 (34.0) **	0.66 (0.43; 0.99) **	0.74 (0.48; 1.13)	94 (91.3) **	2.14 (1.07; 4.27) **	1.84 (0.92; 3.72)
	No	1343 (43.9)	-	-	2539 (83.0)	-	-

Notes: *p*-value ≤ 0.001 * and *p*-value < 0.05 ** through ^ǂ^ Chi-square test and ^¥^ Multivariate analysis. ORA = Odds Ratio Adjusted. Statistical analysis controlled by alcohol use, gender and age. Total = Cocaine [smoked (*n* = 1378) and snorted (*n* = 2633)].

**Table 4:** Physical and interpersonal domestic violence reported by participants.

		Cocaine Smoked			Cocaine Snorted		
		YES ^ǂ^ [*n* (%)]	OR Crude (CI 95%) ^¥^	ORA (CI 95%) ^¥^	YES ^ǂ^ [*n* (%)]	OR Crude (CI 95%) ^¥^	ORA (CI 95%) ^¥^
The drug user has physically assaulted someone in the family	Yes	545 (46.4) **	1.20 (1.03; 1.38) *	1.25 (1.07; 1.45) *	988 (94.1)	1.09 (0.90; 1.33)	-
	No	833 (41.9)	-	-	1648 (82.8)	-	-
The drug user threatened a family member	Yes	365 (40.4) **	0.83 (0.71; 0.97) *	0.94 (0.77; 1.14)	818 (90.5) *	2.32 (1.82; 2.97) *	1.52 (1.14; 2.04) *
	No	1013 (44.9)	-	-	1815 (80.4)	-	-
The drug user assaulted someone else	Yes	256 (39.4) **	0.80 (0.67; 0.96) *	0.86 (0.70; 1.06)	597 (92.0) *	2.70 (1.99; 3.63) *	1.86 (1.32; 2.60) *
	No	1122 (44.6)	-	-	2036 (81.0)	-	-
Threats or violence after a talk regarding the problems related to drug use	Yes	322 (38.8) *	0.76 (0.65; 0.90) *	0.81 (0.67; 0.97) **	747 (90.0) *	2.13 (1.66; 2.73) *	1.50 (1.13; 1.96)
	No	1056 (45.3)	-	-	1886 (80.9)	-	-

Notes: *p*-value ≤0.001 * and *p*-value < 0.05 ** through ^ǂ^ Chi-square test and ^¥^ Multivariate analysis. ORA = Odds Ratio Adjusted. Statistical analysis controlled by alcohol use, gender and age. Total = Cocaine [smoke (*n* = 1378) and snorted (*n* = 2633)].

## Data Availability

Data are available upon request from the author.
